# Functional Assessment of Congenital Radioulnar Synostosis in Children

**DOI:** 10.1055/s-0045-1802671

**Published:** 2025-02-21

**Authors:** Isam Sami Moghamis, Hasan Abuhejleh, Yousef Abuodeh, Hazem Mohamad Nasef, Harris Manova Thanaraj, Ilse Du Preez, Mohamad Alaa Kawas

**Affiliations:** 1 Department of Orthopedics Surgery, Hamad Medical Corporation, Doha, Qatar; 2 Department of Orthopedics Surgery, Farah Medical Campus, Amman, Jordan; 3 Department of Occupational Therapy, Hamad Medical Corporation, Doha, Qatar

**Keywords:** congenital, radioulnar synostosis, management, functional outcome, forearm deformity

## Abstract

**Background** Congenital radioulnar synostosis (CRUS) is a rare condition caused by segmentation failure in embryonic life. The forearm is usually fixed in pronation, affecting the upper limb and hand functions. Treatment ranges from observation to surgical options to restore limb function and prevent disease recurrence. The study aimed to assess the functional outcome of patients with CRUS.

**Methods** We conducted a prospective evaluation of eight patients with CRUS between 2010 and 2020. The data involved history, physical examination, and functional assessment using the ABILHAND-Kids functional scale to determine children's adaptation to this deformity and the indication for surgical management. A control sample of four patients was included in the study for comparison.

**Results** Eight patients with 11 limb deformities were included in the study. The affected limbs' range of motion (ROM) was compared with the ROM of the unaffected limbs and the control patients. There were no statistically significant differences in ROM between the two groups except for fixed pronation deformity of the synostosis ( *p* = 0.0133). The average ABILHAND-Kids score for all children was 38.625 ± 3.021, close to the average score of 42. Only one patient with bilateral involvement underwent surgical correction of the deformity 5 years from the initial presentation.

**Conclusion** CRUS is a rare congenital condition that can affect daily living activities. Surgical correction is rarely indicated and is only preserved for patients with bilateral involvement and significant functional impairment.

## Introduction

Congenital radioulnar synostosis (CRUS) is a rare condition characterized by a fixed relation between the radius and ulna that ranges from moderate to severe fixed pronation secondary to failure of segmentation during embryonic life. 1 2 There have been around 350 reported cases for more than two decades, and despite CRUS being a rare condition, it is one of the most commonly encountered congenital elbow conditions, and it is bilateral in 60 to 80% of the cases. 3

Sandiford first described CRUS in 1793. 4 The elbow is formed from the three cartilaginous condensations representing the humerus, radius, and ulna, which for a short period share a common perichondrium at the fifth week of gestation. These bones formed following a cavitation process that occurs in this perichondrium. At 5 to 6 weeks of gestation, any insult to the cartilaginous analog will fail segmentation and bony fusion of the proximal radius and ulna. 5 Moreover, during this process, the fetal forearm is presented in a pronation position, so the fixed deformity is always found in pronation. 2

Lack of rotation in the forearm can lead to varying degrees of functional disabilities, which can affect the quality of life in performing some activities of daily living, such as eating, catching objects, personal hygiene, dressing, and opening doors. 6 However, the management of such cases depends mainly on the amount of functional disability encountered during activities of daily living. Several reports have described different surgical techniques used in the treatment of CRUS. 7 8 This study presents our observations of eight patients who were presented to our practice and their functional assessment.

## Materials and Methodology

This prospective study, with ID number 11072/11, was approved by our local institutional review board. All patients diagnosed with CRUS and referred to our pediatric orthopedics department between 2010 and 2020 were included in the study. Informed consent was obtained from all parents before the inclusion. The total number of cases diagnosed with CRUS at that period was eight. The study aimed to assess the long-term functional outcome of the deformity.

A detailed medical history was taken from the family, a thorough physical examination was conducted, and X-rays of the affected and normal side were obtained. The Cleary–Omer classification System for CRUS was used for the radiological classification. 2

Functional outcome was assessed using the ABILHAND-Kids, with the assistance of a pediatric and occupational therapist. The ABILHAND-Kids score assesses manual ability as a test focused on the child's difficulty perceived by the child's parents.

Eight patients with 11 congenital radioulnar synostoses were included in the study. Four patients were included as a control group for comparison; they had normal forearm anatomy and function. The assessment sessions were video recorded in two planes to view the deformity position in different activities and evaluate the affected patient's shoulder and elbow compensatory movements.

### Statistical Analysis

The statistical analysis was performed using SPSS (IBM Corp., Armonk, New York, United States). Descriptive statistics, including means and standard deviations, were calculated for continuous, normally distributed variables, while frequencies and percentages were used for categorical variables. The normality of continuous variables was assessed visually using histograms. For comparisons between groups, independent two-sample *t* -tests were employed for normally distributed data, and the Mann–Whitney *U* test was applied when data did not meet normality assumptions. A significance level of 0.05 was used, and effect sizes were calculated to measure the magnitude of differences.

## Results

Eight patients, one girl and seven boys, were included in the study, with 11 forearm deformities. Two patients had left-sided deformities, two had right-sided deformities, and the remaining had bilateral involvement. The mean age at presentation was 6 years (72 ± 34.2 months). According to Cleary and Omer's radiological classification of the anomalies, 6 forearms had type 1 deformity, 2 had type 2 deformity, another 2 limbs had type 3 deformity, and only 1 deformity was of type 4.

**Fig. 1:**
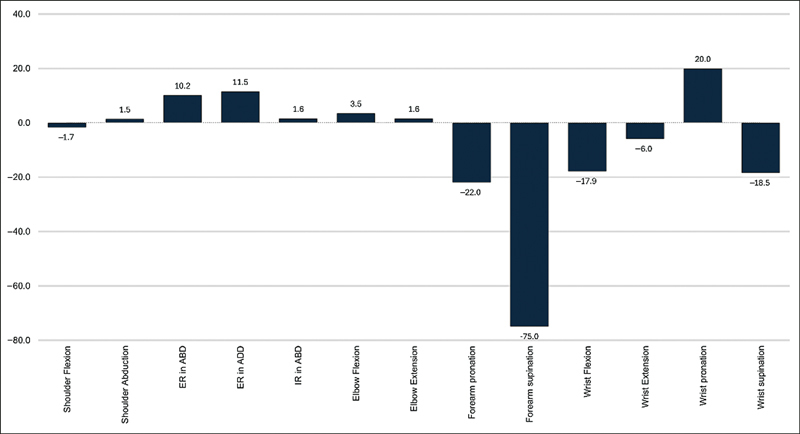
Bar chart for mean differences in range of motion (ROM) for elbow, forearm, and wrist functions.

The affected limbs' range of motion (ROM) was compared with the unaffected limbs and control patients ( Table 1 ). There were no statistically significant differences in ROM between the two groups except for fixed pronation deformity of the synostosis ( *p* = 0.0133). The mean differences for the functions are presented in Fig. 1 . The average ABILHAND-Kids score for all children was 38.625 ± 3.021, close to the average score of 42.

**Table 1:** Demographics and functional assessment

	Affected limbs		Unaffected limbs and control group		Mean difference	95% CI (LCI, UCI)	*p* -value
Number	11		13				
	**Mean**	**SD**	**Mean**	**SD**			
Age (mo)	72	34.2	78	48.7			0.7791
**Shoulder ROM**							
Flexion	172.3	20.9	174.0	13.4	−1.7	−16.9 to 13.5	0.8710
Abduction	110.5	9.6	109.0	12.4	1.5	−7.8 to 10.8	0.7945
ER in abduction	108.2	10.8	98.0	14.8	10.2	−0.7 to 21.1	0.1397
ER in adduction	79.5	15.2	68.0	24.9	11.5	−5.7 to 28.7	0.2684
IR in abduction	68.6	10.0	67.0	21.7	1.6	−12.4 to 15.6	0.8392
**Elbow ROM**							
Flexion	149.5	19.3	146.0	19.5	3.5	−13.0 to 20.0	0.7424
Extension	−1.4	8.1	−3.0	4.5	1.6	−4.1 to 7.3	0.8858
**Forearm ROM**							
Pronation	45.0	19.6	67.0	21.9	−22.0	−39.6 to −4.4	0.0133
Supination	0	0	75.0	26.0	−75.0	−90.0 to −60.0	
**Wrist ROM**							
Flexion	68.1	28.5	86.0	5.5	−17.9	−36.0 to 0.2	0.066
Extension	80.0	17.9	86.0	5.5	−6.0	−17.6 to 5.6	0.4878
Pronation	59.0	28.9	39.0	37.5	−20.0	−8.1 to 48.1	0.3006
Supination	27.5	29.2	46.0	32.9	−18.5	−44.8 to 7.8	0.3116

Abbreviations: CI, confidence interval; ER, external rotation; IR, internal rotation; LCI, lower confidence interval; ROM, range of motion; SD, standard deviation; UCI, upper confidence interval.

Only one patient in our series had undergone surgical correction of the deformity because he had bilateral involvement, fixed pronation deformity of 70 degrees, and severe functional limitation of the upper limbs that affected his school performance and daily activities. His surgery was carried out 5 years after the first presentation. He underwent derotational osteotomy of the radius and ulna. The dominant forearm was fixed in 15 degrees of forearm pronation, that is, functional position. At the same time, the other nondominant hand was fixed in 30 degrees of pronation. Postoperatively, he had no surgery-related complications, and the patient was doing well during the follow-up. His functional limitation was improved, he could better perform daily activities, and his school performance improved.

## Discussion

Objective measurement of functional status in children with CRUS is essential for management and treatment planning. The described forearm deformity affects several patients' daily activities. Patients cannot accept coins and small objects in open palms and have difficulty holding plates and cups or combing their hair. Chopstick use may be limited in Asian countries, and patients report difficulty manipulating smartphones. 9 10 However, only a few articles have reported pain and dissatisfaction in adult life, and no study has shown how activities change or deteriorate with age. 11

Patients with CRUS have difficulties in daily activities that require forearm supination. They usually compensate for the missing supination with shoulder adduction and the fixed forearm pronation with shoulder abduction. 12 Kasten et al, in a three-dimensional motion analysis of compensatory movements of the shoulder and elbow, found that patients with CRUS could perform activities of daily living with the help of compensatory movement of the shoulder and elbow. 13 In our study, there was an increase in the shoulder ROM, mainly external rotation in abduction and adduction, but no significant difference was observed compared to normal limbs. However, in cases of severe hyperpronation deformity, this compensatory mechanism fails. Furthermore, the two groups had no difference in elbow flexion and extension, but Shinohara et al reported two cases of CRUS with decreased carrying angle and deficit of elbow extension. 14

ABILHAND-Kids is a scale that measures a child's manual performance of 21 daily activities as perceived by the parents. It is suitable for children with unilateral and bilateral impairments, as assessing bimanual performance in children with congenital hand deformities provides a more accurate measurement of functional limitations. 15 In our series, the average ABILHAND-Kids score was near the control group's average. This reflects that CRUS rarely affects the bimanual hand function, even in cases of bilateral involvement, and this might be due to the maintained normal ROM of the elbow, wrist, and shoulder joints to compensate for the deficit forearm pronation and supination. However, bimanual involvement had the most impact on the measured scores. Despite the good function observed in these kids, the parents always ask for treatment to restore normal motion. Unfortunately, there are no clear guidelines or evidence-based rules for managing this deformity.

The treatment of CRUS varies from nonoperative management to different surgical procedures intended to improve function. For example, Kepenek-Varol and Hoşbay reported improved functional outcomes following short-term hand therapy in the case of CRUS. However, the ROM was not affected by this treatment modality. 9 Over time, surgical indications for CRUS have been debated. Some authors believe that surgeries are rarely indicated and that functional deficit is the only indication for surgery. 2 On the contrary, some authors suggested that a fixed pronation of ≥60 degrees indicates surgical intervention. 6 16 At the same time, Yammine et al recommended surgical intervention when there is bilateral involvement. 17 Furthermore, cultural habits and customers can also affect surgical indications. 18 Our patient who underwent surgical intervention had bilateral involvement; moreover, he had a significant functional disability that affected his school performance. These causes can justify the necessity of surgical intervention.

Derotational osteotomy is a surgical method for treating CRUS. It was developed to derotate the forearm to a more functional position, but it does not restore the active ROM of the forearm. Another surgical option is the mobilization procedure, which tends to remove the synostosis and reconstruct the proximal radioulnar joint (PRUJ). Unfortunately, this treatment has a high postoperative recurrence of the synostosis. 19 Because of this drawback, surgeons have continuously searched for a better mobilization technique. In 1998, Kanaya et al reported a good outcome with the combination of the synostosis resection and free vascularized fascial–fat graft interposition. 20 However, the lateral producer often finds it difficult to realign the radial head properly, leading to anterior or posterior radial head dislocation. 6

Our study's main limitation is the small patient sample size. Further study with a bigger sample size can better assess the disease and indicate the need for surgical deformity correction.

## Conclusion

CRUS is a rare congenital condition in which the patient has a fixed pronation deformity secondary to the failure of segmentation between the radius and ulna in embryonic life. Short-term hand therapy may improve functional outcomes. However, surgical correction is indicated mainly for bilateral involvement with forearm hyperpronation deformity and if there is significant impairment of daily life activities. Surgical correction of this deformity has a favorable outcome, especially in patients with substantial functional difficulties.
